# Chemicals, Climate, and Control: Increasing the Effectiveness of Malaria Vector Control Tools by Considering Relevant Temperatures

**DOI:** 10.1371/journal.ppat.1003602

**Published:** 2013-10-03

**Authors:** Katey D. Glunt, Justine I. Blanford, Krijn P. Paaijmans

**Affiliations:** 1 Center for Infectious Disease Dynamics and Department of Biology, The Pennsylvania State University, University Park, Pennsylvania, United States of America; 2 GeoVISTA Center, Department of Geography, The Pennsylvania State University, University Park, Pennsylvania, United States of America; 3 Barcelona Centre for International Health Research (CRESIB, Hospital Clínic-Universitat de Barcelona), Barcelona, Spain; The Fox Chase Cancer Center, United States of America

## Introduction

Malaria vector control currently relies almost exclusively on killing adult mosquitoes with chemical insecticides. Insecticide-treated nets (ITNs), long-lasting insecticide-treated nets (LLINs), and indoor residual sprays (IRS) aim to repel, disable, and/or kill mosquitoes on contact. While these tools have proven to be extremely successful in reducing disease incidence and mortality [1], insecticide resistance is on the rise and a resurgence of malaria is feared [2]. To mitigate the effects of resistance, the development of new insecticides and formulations for use in LLINs and for IRS remains a research priority [3]. In this paper we argue that, to increase the effectiveness of the chemical arsenal available, we need to consider the relevant microclimatic conditions in which these tools are deployed. We will discuss how temperature in particular can interact with the conventional use of chemicals within houses, and broaden our discussion to consider its potential influence on the use of semiochemicals to lure mosquitoes to traps.

## Test Temperatures Are Higher Than Mosquitoes Typically Experience in the Field

The World Health Organization Pesticide Evaluation Scheme (WHOPES), which promotes and coordinates the testing and evaluation of pesticides for public health, specifies laboratory conditions in their guidelines for testing mosquitocidal compounds and products. The recommended temperatures for phase I trials are 25±2°C for testing of LLINs [4] and 27±2°C for IRS and treated bednets [5].

**Figure 1:**
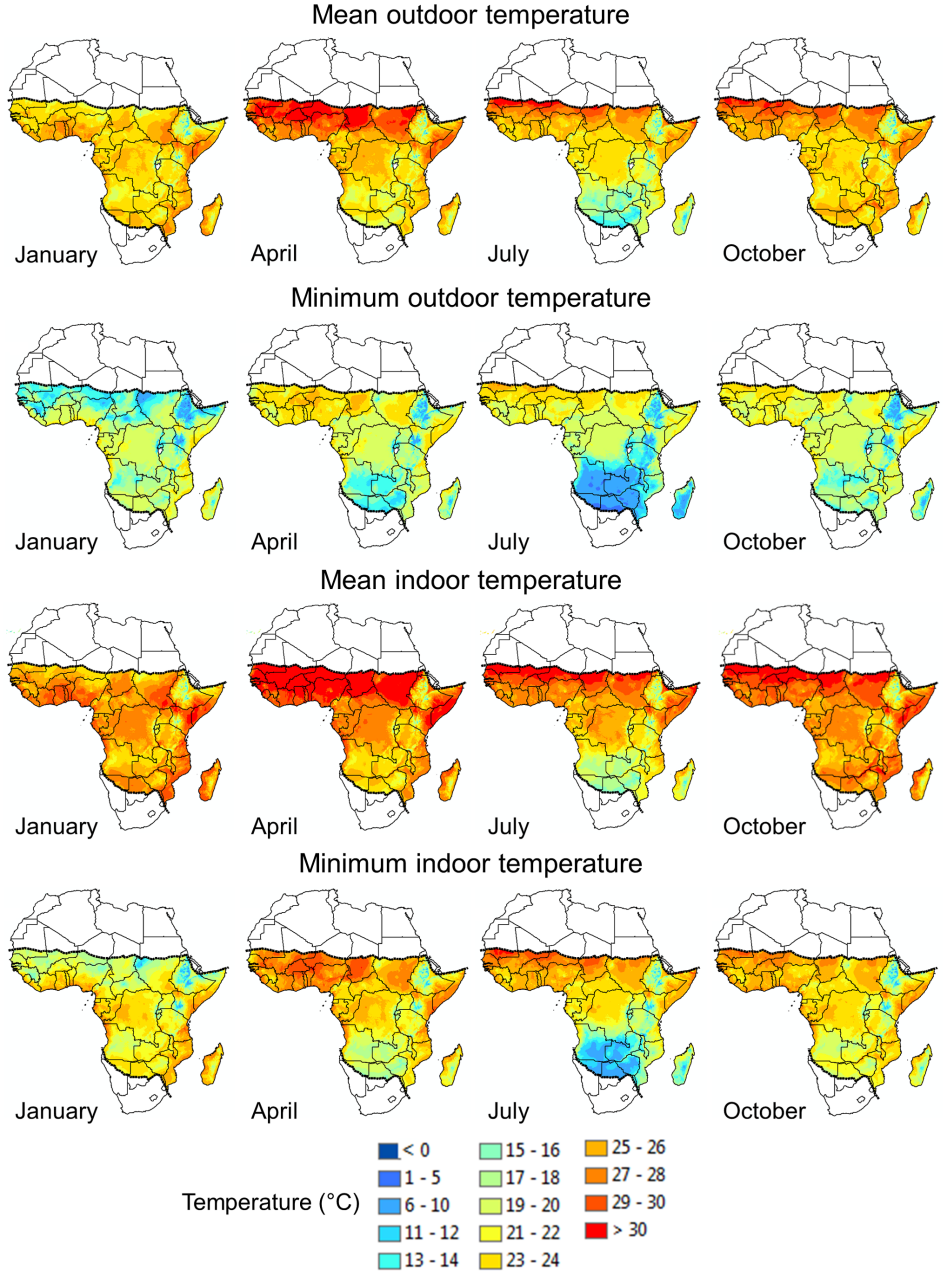
Monthly mean and minimum outdoor and indoor temperatures throughout Africa for January, April, July, and October. *Outdoor monthly mean (top row) and minimum (second row) temperatures.* Temperature surfaces were generated by interpolation using weather station data collected between 1960 and 1990. For areas where data records were limited, such as in the Democratic Republic of the Congo, the time period was extended to 2000 (see [45] for details). The current geographical limits of malaria transmission are demarcated by the dotted lines. *Indoor monthly mean (third row) and minimum (bottom row) temperatures.* Indoor temperature estimates were determined using regression equations that capture the relationship between indoor and outdoor temperatures at different elevations. These regressions were used to convert the outdoor temperature surfaces to matching estimates of indoor temperatures (see [46] for more detailed information).

Though temperatures are standardized to improve the reliability and reproducibility of the tests, the ranges chosen are only observed in small geographical areas of sub-Saharan Africa, mainly directly south of the desert (Figure 1, top row). In most malaria transmission settings, the observed mean temperatures range from approximately 18 (cooler highland areas) to 26°C.

Even more importantly, many vectors of malaria are actively host seeking and blood feeding from dusk until dawn [6], when temperatures are considerably lower than the daily mean. Nighttime minimum temperatures of around 25°C are mostly limited to small areas directly south of the Sahara; in general, minima range from about 13 to 22°C in most malaria transmission zones, depending on season and location (Figure 1, second row).

Temperatures inside houses are generally a few degrees Celsius warmer than those recorded outdoors, and mean indoor temperatures around 25°C can be observed in larger geographic areas (Figure 1, third row). However, indoor minimum temperatures remain well below 25–27°C (Figure 1, bottom row) with large areas experiencing <22°C. It is under these environmental conditions that a mosquito is searching for and biting new hosts.

## Susceptible Mosquitoes Could Be More Resistant during Cooler Nighttime Periods

**Figure 2:**
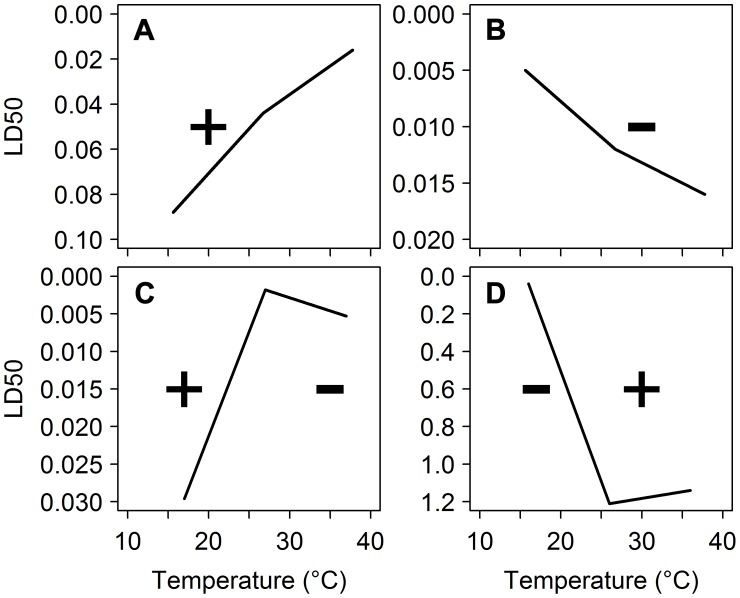
Temperature coefficients of deltamethrin against different insect species. Toxicity (median lethal dose) of deltamethrin to (A) *Heliothis virescens* (µg/g) [17], (B) *Trichoplusia ni* (µg/g) [17], (C) *Chilo suppressalis* (µg/insect) [16], and (D) *Triatoma infestans* (ng/insect) [47]. Note that the Y-axis is inverted to visualize the temperature coefficient (TC). If the dose required to kill 50% of insects decreases as temperature increases, the insecticide has a positive TC, indicated by +. Negative TC indicated by −.

The insecticides used in public health for vector control kill mosquitoes by interfering with nervous system function. But metabolic activity [7], which is involved in degradation of insecticides, and nervous system sensitivity [8] are highly temperature-dependent. As mosquito body temperature changes with its surroundings, environmental temperature has the potential to influence the toxicity of insecticides. This effect is quantified by measuring the temperature coefficient (TC) of an insecticide (Figure 2). A positive TC indicates that an insecticide becomes more toxic as temperature increases; insecticides with a negative TC kill more insects at lower temperatures. Pyrethroids, the dominant insecticide class currently used for malaria control, and DDT, the only organochlorine permitted for IRS, commonly exhibit a negative temperature coefficient. Therefore, in theory, they should perform better under cooler nighttime conditions. On the other hand, carbamates and organophosphates (two and three out of the 12 recommended compounds for IRS, respectively) generally have a positive TC, and may be less efficient under these conditions.

Exceptions to these general TC rules, however, are common. Whether the TC is positive or negative can depend on the insect (e.g., species, developmental stage, age, sex), the chemical tested (e.g., formulation, substrate, dose, duration of exposure) and testing conditions (e.g., temperature range, humidity, time of day). For pyrethroids, the only insecticides currently used on ITNs and LLINs [9], [10] and the dominant insecticide class in IRS [11], a strictly negative relationship with temperature is not always observed. Type II pyrethroids, which have an α-cyano group on the phenoxybenzyl moiety [12], tend to violate this rule. For example, deltamethrin and cypermethrin, which are used in 11 out of the 13 (or 85%) LLINs, have been observed to have positive TCs for mortality in several insect species (Figure 2A, C, D, [13]–[17]). If the same phenomenon applies to malaria mosquitoes, only DDT and two LLINs (those treated with permethrin, a type I pyrethroid) will be most effective during the cooler nighttime periods when a mosquito is active: all other recommended interventions could be less effective at killing vectors.

To the best of our knowledge, there are only two studies that examined the effect of temperature on the toxicity of insecticides on susceptible, adult malaria vectors. Hodjati and Curtis [18] observed a bimodal relationship between temperature and toxicity for permethrin, a type I pyrethroid, against *Anopheles stephensi*, a primary malaria vector in India (negative between 16 and 22°C, but positive between 22 and 37°C). Over the same range of temperatures, *An. gambiae*, a major malaria vector in sub-Saharan Africa, displayed a consistently positive TC. This indicates that malaria mosquitoes may not follow the general temperature-toxicity rules. The second study [19] saw a negative TC for DDT and a positive TC for the organophosphate diazinon when *An. stephensi* was exposed to insecticide residues between 20 and 30°C.

While vector control chemicals are typically applied at concentrations meant to overwhelm variation in susceptibility, evidence from the field shows that the ability of LLINs or IRS to kill mosquitoes can decrease rapidly over time after initial deployment. Although LLINs should retain their insecticidal activity for at least three years under field conditions [20], the mosquitocidal activity of several LLINs is reduced on much shorter time scales [21], [22]. The activity of IRS compounds can decline significantly within the first few months after spraying due to, for example, variation in building materials [22], [23] or in the spraying technique of individual applicators [24]. Thus, there could be periods prior to IRS retreatment or redistribution of new LLINs during which the loss of efficacy from chemical, operational, or environmental factors could be exacerbated by using chemicals that are even less effective under variable temperature conditions.

With approximately 34 dominant anopheline vector species in the world [25], and a variety of recommended chemical products on the market, this lack of data represents a critical gap in our understanding. Although current tools do kill mosquitoes and reduce malaria risk, a better understanding of chemical temperature coefficients could affect the chemical toolbox in two ways: first, it could increase the number of chemicals available for control. By testing insecticidal performance under standard laboratory conditions (25–27°C), there is the possibility that we currently eliminate compounds in the testing phase—especially those with a strongly negative TC—that may perform very well in the field. Second, without information about their action at different temperatures, we may deploy chemicals that will be less efficient than we expect under actual field conditions. Investigating the performance of our vector control tools under different temperature conditions will augment our ability to select the most efficacious tool for a given environment. For insecticidal control of pests in crop systems, it has been acknowledged that knowing a product's temperature coefficient enables pest managers to select a product that is efficacious under the prevailing environmental conditions [26]–[28].

## Resistant Mosquitoes May Be More Resistant in the Laboratory

Insecticide resistance is one of the greatest threats to the success of malaria control and elimination campaigns. The WHO currently recommends that the level of resistance in mosquito populations be evaluated at 25±2°C [29]. As with susceptible insects, the mortality of resistant insects can increase or decrease with temperature (e.g., [30], [31]). Hodjati and Curtis [18] showed that resistant *An. stephensi* mosquitoes were more susceptible to permethrin at 16 and 37°C, compared to 22 and 28°C, where nearly all mosquitoes survived the exposure. In resistant *An. gambiae*, as in the susceptible strain, susceptibility increased with temperature. This suggests that quantifying resistance under relatively high temperature conditions in the laboratory will not necessarily inform us to what extent a chemical intervention is still effective in the field.

## Efficiency of Other (Semio)chemical Interventions Will Also Depend on Environmental Temperature

There is growing evidence that the widespread use of LLINs and IRS is reducing mosquito activity indoors and can drive vector-species composition changes or host-species switching behavior to increase outdoor biting [32]. Alternative interventions that specifically target outdoor biting are needed. One approach is to use chemical compounds to trap or repel mosquitoes, thereby reducing the number of mosquito bites to human hosts. There are reasons to expect that the effectiveness of such odor-baited traps could be affected by environmental temperature.

For odor-baited traps to work, a mosquito needs to detect the odor plume and follow it back to the source. The number of odor molecules of a compound in the gas phase will be reduced when temperatures decrease (see example in [33]). Simply put, there will be less for a mosquito to smell when it is cooler outside. Additionally, odor plume dynamics depend on the stability of the atmosphere, which depends in part on temperature [34]. Although adding a heat source could regulate the release of molecules from a trapping device, the resulting odor plume can be expected to behave differently under cool nighttime conditions than it would under warmer laboratory conditions. In addition, temperature affects several physiological processes involved in insects' odor reception [35], [36]. Lower temperatures can reduce response distance and specificity [37], but also directly impact insect flight behavior by reducing flight speed [38].

So, although these traps seem to work in the field [39], cooler field temperatures may reduce trap efficiency, which has been shown for adult plum curculios, *Conotrachelus nenuphar* [40]. At present, the behavioral responses of mosquitoes to chemical cues in olfactometers are evaluated at standard insectary conditions, around 26–27°C [41], [42], and there are no WHO guidelines for testing such devices. Again, as malaria mosquitoes host seek and bite only during the cooler evening and night, there might be room for improvement when the actual microclimate observed in the field is considered during laboratory trials.

## Conclusions

Chemicals are powerful tools in the control of malaria and other vector-borne diseases such as dengue, leishmaniasis, and Chagas disease [43]. Given that temperature has the potential to affect the toxicity of chemicals used for ITNs, LLINs, and IRS, as well as to alter chemical release from and mosquito response to odor-baited traps, candidate chemicals need to be evaluated under relevant climatic conditions. For the initial development of chemicals to be used in the fight against malaria, we suggest that testing recommendations, currently at 25 to 27±2°C, should include a range of temperatures: 15, 20, 25, and 30°C. Such a change would provide valuable information about how mosquitoes and chemicals will interact under natural field conditions, therefore allowing us to develop more effective tools in the laboratory and to select the tools most likely to be effective in a given local environment. As insecticide resistance monitoring in the field is frequently carried out in areas where malaria is endemic (or epidemic), and these areas are often low-income countries, we suggest adding one additional temperature for these tests: 20°C. This change will give us a better understanding of how well the chemicals currently being used are working to control night-biting vectors. In areas where insecticide resistance has been detected in the mosquito population, such knowledge could be especially valuable. By applying a mixture of chemicals, which may also counter or postpone the development of insecticide resistance in mosquito populations to chemicals used on ITNs, LLINs, and in IRS [3], a given regimen could be efficient across different thermal environments, or in environments with a wide thermal envelope [44]. We believe that considering the temperature coefficient of chemicals from the outset of testing will increase the effectiveness of the chemical toolbox for malaria vector control.
